# The Gender Affirmative Treatment Model for Youth with Gender Dysphoria: A Medical Advance or Dangerous Medicine?

**DOI:** 10.1007/s10508-021-02232-0

**Published:** 2021-11-22

**Authors:** Alison Clayton

**Affiliations:** School of Historical and Philosophical Studies, Faculty of Arts, The University of Melbourne, Melbourne, Victoria 3010 Australia

## Introduction

A knowledge of the history of medicine enriches our thinking about contemporary medical practices. The twentieth century saw many medical advances. It also saw multiple examples of what may be called dangerous medicine. Such medicine is invasive, risky, and lacking a rigorous evidence base, but is enthusiastically embraced and celebrated by members of the medical profession and the public. Then, with the passage of time, such medicine is viewed with more scepticism. It is recognized as not being as beneficial as claimed and as causing more harm than acknowledged. It comes to be mostly seen as misguided, occasionally even criminal. In this Letter, I use a historical frame to background a discussion of the gender affirmative treatment approach for youth with gender dysphoria (GD youth), particularly focusing on masculinizing chest surgery. I ask: Is this approach a medical advance or is it a contemporary example of dangerous medicine? My hope is that the ideas expressed in this Letter will helpfully contribute to the debate about this complex and controversial area of medicine.

Braslow (1997) discussed the history of twentieth century psychiatry as practiced in California state mental hospitals. He described how doctors can transform the harmful into the “therapeutic” with their implementation of invasive “treatments” on patients in their care. He gave an illustrative example of how a “woman’s body provided multiple sites for surgical interventions” that aimed to extinguish “pathological behavior.” He described a 28 year-old Californian “housewife,” Rose, admitted as an involuntary patient in 1944. In 1949, her disturbed mental state and uncooperative behavior led to her receiving a radical prefrontal lobotomy. A year later, her “amorous” behavior led her doctor to order a clitoral cauterization. The day following this “minor procedure” it was noted she continued masturbating and a second clitoral cauterization was performed. Rose then developed the habit of biting people, the solution for which was teeth extraction (pp. 166–168).

This brief account of Rose evokes a range of emotions. I feel horror, sadness, anger, and fear. Why do I feel these emotions? I am a human and a woman. I have had the experience of being a patient in doctors’ hands, relying on their expertise, care, and conscientiousness. It is the stuff of nightmares to be totally dependent on physicians, who, even if well-meaning, are implementing ineffective, harmful, or punitive interventions. My emotional reaction to Rose’s story is made even more complex, because I am also a psychiatrist. Therefore, part of my horror in reading this history is that I am identified with those physicians implementing such “treatments.” I ask myself: How would I have treated patients as a psychiatrist in the 1940s? Would I have done what Rose’s psychiatrist did?

Another celebrated historical treatment was malaria fever therapy, which entailed deliberately inducing a malarial illness in patients suffering from general paralysis of the insane (GPI). GPI was a severe form of neurosyphilis and was a frequent cause of admission to the early twentieth century psychiatric institutions. Although risky, malaria therapy was claimed to be successful in curing some patients from this usually fatal disease. It was used from the 1920s until the 1950s, gradually being superseded by penicillin (Davis, 2008). The British psychiatrist Shaw (1929) offered a dissenting view on this celebrated treatment. He imagined a history of psychiatry in the year 2500 making a “curious and interesting commentary” about the people in the early twentieth century who engaged in an “organized attempt to deplete their insane population by infecting them with a disease known as malaria” (p. 15).

It is a curious experience reading Shaw’s words in 2021: past, present, and future become entangled. Contemporary historians are critical of “Whiggish” histories of medicine, which stand on a moral high ground to criticize the “bad old days” (Lerner & Caplan, 2016). However, others warn against using historical context as an exculpatory “moral shelter” (Reverby, 2018; Sadowsky, 2017, p. 68). What happens if we turn the tables and ask: How would historical physicians report on medicine circa 2021, and how will it be reported 100 years hence? Such a thought experiment may help expand our thinking about contemporary medical practices. Here, I apply it specifically to the practice of masculinizing chest surgery for GD youth.

## Twenty-First Century Medicine: Masculinizing Chest Surgery for Youth with Gender Dysphoria

Previously a rare phenomenon, increasing numbers of young people are presenting to clinicians with gender dysphoria. The largest group are natal female adolescents, many with a history of psychiatric illness or neurodevelopmental disorders (Tollit et al., 2021). Among this population, there is a “high demand” for surgical removal of breasts (Kuper et al., 2021; Telfer, 2019, p. 14) and this is being undertaken as routine treatment in patients as young as 13 (Olson-Kennedy et al., 2018). A number of clinicians argue that this surgery is an evidence-based intervention that improves mental health outcomes and that it is discriminatory for it not to be available (McDougall et al., 2021; Mehringer et al., 2021; Olson-Kennedy et al., 2018; Telfer, 2019).

Before discussing the evidence base, some explanations on terminology are required. The studies that I discuss mostly report on surgery for individuals less than 21 years old. A number of terms are used to describe this population, including “adolescents,” “minors,” and “youth.” I use the authors’ own terms when discussing their papers; otherwise, I use the term “youth.” “Chest dysphoria” is a recently created term meaning the discomfort with one’s breasts. The term “breasts” is largely absent in these academic publications as it “may cause distress for transgender males” (McDougall et al., 2021) and this seems part of broader pattern of removing this term from clinical language (Lehmann, 2021). The surgical terminology is also changing. “Mastectomy” is being replaced by terms such as “chest surgery,” “top surgery,” “masculinizing chest surgery,” or “chest contouring” (Cohen et al., 2019; McDougall et al., 2021; Mehringer et al., 2021; van de Grift et al., 2016).

There are only a handful of published studies focusing on the potential benefits of masculinizing chest surgery in youth. Marinkovic and Newfield (2017) published what they claimed was the first study reporting data on chest surgery in young adolescents. In this retrospective observational study of 14 postsurgical youth, nine of whom were under 18 years old, participants rated satisfaction with the aesthetic outcome of the procedure on a Likert-type linear scale. All reported high aesthetic satisfaction and most self-reported low complication rates and improvement in mood. The postsurgical clinical follow-up, 1.6 years on average, was described as mostly “uneventful.” However, one participant, seven months after surgery, requested help to detransition and was subsequently lost to follow-up.

Olson-Kennedy et al. (2018), stating there were no prior “data documenting the effect of chest surgery on minors,” undertook a cross-sectional retrospective survey of 68 postsurgical transmasculine youth (72% of the eligible postsurgical population). In 49%, the surgery had been undertaken at younger than 18 years of age, with the youngest being age 13 and the oldest age 24. At the time of the survey, only 14% of the participants were more than 2 years post-surgery. The postsurgical participants were compared with a convenience and non-matched comparison sample of nonsurgical transmasculine youth. The outcome, chest dysphoria, was measured with an unvalidated scale and indicated that the postsurgical participants had less chest dysphoria than the nonsurgical participants. Another notable finding was that testosterone use was associated with increased chest dysphoria. It is important to note that, a few years prior to this study, Olson-Kennedy (2015) had already been promoting chest surgery for minors, describing it as easy, safe, available, and “absolutely life-saving.” This last claim seems contradictory to the 2018 paper which stated there were no previous data on chest surgery in minors. Olson-Kennedy (2015) also stated that “full gender-affirming surgery” in minors was “on the horizon” and noted “the difficulty of genital surgery is that it is surgical sterilization and people get super worked up about that…it is a barrier we have to over-come and I think we are going to.” It seems this barrier is already being over-come, as it has been reported that in the United States genital surgery is being undertaken on GD minors, as young as 15 years old (Milrod & Karasic, 2017).

Kuper et al. (2020) reported on short-term body dissatisfaction and mental health outcomes in 148 adolescents receiving gender affirmative medical treatments. Out of this group, 15 “affirmed males” obtained chest surgery, at an average age of 17.1 years (the youngest being 15.2 years). Kuper et al. found no significant correlation between chest surgery and psychological outcomes.

Lastly, Mehringer et al. (2021) reported a cross-sectional qualitative study of chest dysphoria in transmasculine youth. They selected 35 participants, of whom 30 completed the study. Of these, 47% had undergone chest surgery (on average 19 months previously), and 53% had not had surgery. Mehringer et al. concluded that the postsurgical cohort experienced “tremendous” benefits in chest dysphoria and a range of psychological outcomes; however, in my opinion, they did not provide enough detail for the reader to make an informed judgement regarding this latter claim.

To my knowledge, this is the main published evidence base for the potential benefits of masculinizing chest surgery in GD youth. Importantly, it appears that the chest surgery reported on in these studies was not undertaken as part of any human research ethics committee (HREC) approved clinical trial–as the surgery was undertaken prior to or separate from the research.

## Reflections on the Evidence Base for Chest Surgery for Gender Dysphoric Youth

I now return to my previously posed question: How would historical medical practitioners comment on this surgical intervention? I imagine Shaw, in response to hearing about the surgical sterilization of vulnerable youth, drily commenting that the eugenic agenda of early twentieth century psychiatry and medicine might still be operative in 2021. Shaw might note that removing the term “breasts” from our language parallels early twentieth century English and American society, which was full of euphemisms to avoid distressing women. Freeman (1961), an American psychiatrist, whose lobotomy patients included distressed adolescents as young as age 12, might exclaim “and you judge me?!” Historical physicians would likely laugh at twenty-first century physicians’ claims that contemporary medical practices are based on rigorous evidence compared to the unscientific medicine of the past.

Indeed, many of the criticisms made about the inadequate evidence base of discredited historical treatments can be leveled against chest surgery for GD youth. The studies have significant methodological limitations which mean they are at critical risk of bias and cannot show that chest surgery is causally associated with short-term improved mental health outcomes. They do not provide any information on long-term outcomes and regret rates. Limiting features include the following: small and select samples; cross-sectional assessment; uncontrolled case series or comparative studies with non-matched control groups; high drop-out rates; short-term follow-up only; unvalidated measurement scales; and a failure to account for the impact of co-interventions and the placebo effect. Those advocating for chest surgery in youth make strong claims regarding the evidence base for this surgery in adults. However, these are also limited by similar shortcomings (Cohen et al., 2019). Furthermore, the research is notable for its failure to assess a role for psychological interventions which could be utilized, as a least harm intervention, until maturity is reached. One study, on adults, actually did discuss a possible role for psychological help with “body acceptance,” but it only came at the conclusion and as a suggestion for managing body image problems that remained post-mastectomy (van de Grift et al., 2016). In my opinion, it is surprising that clinicians and researchers claim chest surgery for GD youth is an evidence-based intervention, rather than acknowledging it is an experimental treatment that requires more rigorous and HREC approved research.

How will masculinizing chest surgery for GD youth be viewed by future generations? The enthusiasm for it, despite the lack of a rigorous evidence base, suggests that it may be seen as another example of what Valenstein (1986) called “great and desperate cures.” Valenstein noted that uncontrolled therapeutic experimentation in medicine is common and has the potential to inflict serious harm in the name of progress. He argued that we need to draw lessons from history to help prevent the recurrence of such dangerous medicine. He also emphasized the role of personal ambition. The originators of the lobotomy, Moniz, and malaria therapy, Wagner-Jauregg, reached the pinnacle of medical ambition, each being awarded a Nobel Prize (ibid; Wagner-Jauregg, 1927). Valenstein argued for more regulation of innovative surgery, but also thought that such controls would be resisted “with argument that they will hamper progress and deprive desperate patients of help” and that “randomized control studies are unethical” (Valenstein, 1986, p. 297). This certainly parallels the arguments of those enthusiastically advocating for surgery for GD youth. Valenstein’s view was that “the negative effect of reasonable regulation was exaggerated, especially when compared with the cost of uncontrolled experimentation” (ibid).

## What Can Historical Examples Teach Us about Possible Contributing Factors to Dangerous Medicine?

What factors, aside from the lack of a rigorous evidence base, might contribute to dangerous medical practices? Can history inform us anything about what might lead physicians to over-enthusiastically and prematurely embrace risky and unproven treatments? History suggests there is most likely a complex interplay of multiple factors, and I discuss some of these in this section. In the subsequent section, I return to discuss the gender affirmative treatment approach for GD youth and highlight some themes that may suggest some parallels with this history.

For physicians, “great and desperate” treatments could lead to “name and fame” and inculcate a sense of heroic doctor. For example, at the end of the nineteenth century, general paralysis of the insane confounded physicians’ therapeutic potency, leaving them just contemplating death “like men in a boat about to be swept over a fall, paralyzed with despair” (Godding, 1897). However, malaria therapy changed that and gave doctors a sense of potency and their patients a sense of hope (Braslow, 1997, p. 94; Grob, 1994, p. 180). Today, the efficacy of historical malaria therapy for GPI is questioned (Austin et al., 1992; Davis, 2008, p. 189; Frankenburg & Baldessarini, 2008; Scull, 2015). However, it certainly was effective in bolstering psychiatrists’ status. Ellery, the psychiatrist who introduced malaria therapy to Australia, described that it allowed psychiatrists to be considered “bona-fide clinicians,” who were no longer shunned as asylum medical officers just practicing “black magic” and “voodooism,” but instead “invited to medical meetings and listened to with interest” (Ellery, 1956, pp. 153–154).

An uncritical press portrayed the physicians implementing these treatments as bold medical heroes with the courage to take “desperate remedies” required to cure “desperate ills” (Hurn, 1998, p. 226). Malaria therapy was widely reported in the press with dramatic headlines and celebratory accounts. For example, in Australia, an article entitled “Doctor Mosquito” (1926) promoted malaria therapy’s “wonderful” benefits. In leading international newspapers, such as the *New York Times,* lobotomists were described as simply cutting out the “worry center” of the mentally ill, just as easily as they could remove an “appendix” or an “infected tooth” and bringing the patients a world “radiant with sunshine and kindness” (Harrington, 2019, p. 69; Pressman, 1998, p. 185; Scull, 2015, p. 317). These glowing portrayals, along with Nobel Prizes, gave these treatments a powerful authority, contributing to their rapid and widespread adoption by the medical community and leading patients and their families, including the rich and powerful, to seek them out (Diefenbach et al., 1999). Harrington (2019) reported on the tragic story of Rosemary Kennedy (President Kennedy’s sister) who had a mild intellectual deficit. She was impetuous and the family feared an “embarrassing” pregnancy might result. In 1941, when she was 20, her father arranged for Freeman to lobotomize her. The operation was a disaster and Rosemary was institutionalized for the rest of her life.

Physicians can be confronted by patients whose illnesses, behaviors, and physicality undermine Western ideals of masculinity, femininity, and heterosexual norms. It may be that these patients are particularly at risk of dangerous medicine. General paralysis of the insane was a disease predominantly diagnosed in men in the prime-of-life. It was intimately linked with masculinity and sexuality, and deeply confronting to early twentieth century Anglo-European ideals of manhood. Men could turn from being rational and responsible citizens to filthy, raving, and violent–Mr Hyde, from *The Strange Case of Dr Jeckyll and Mr Hyde,* could have been GPI personified (Swain, 2018). As the disease progressed, young men were reduced to “intellectual drivel and physical wreck,” undergoing a “loathsome bodily decay” (Godding, 1897). In the early twentieth century, GPI’s causation was definitively linked to syphilis and it then became linked to sexual morality. “Bad women” (prostitutes or promiscuous) were condemned for infecting men with venereal disease and, in various jurisdictions, criminal laws were introduced to detain such women. A World War I poster warned the soldiers against sexual liaisons: “A German Bullet is Cleaner than a Whore” (Brandt, 1985, p. 101). Given these attitudes, malaria therapy may have functioned, as did many previous treatments for syphilis, at least unconsciously, as a form of punishment as well as a treatment (Brandt, 1985, p. 12; Quétel, 1990, p. 59). A summary by the well-known popularizer of medical knowledge, de Kruif, suggests this underlying attitude: “Give your paretics the right kind of malaria…and though it burned them, the whole bodies of these paralytics seemed cleansed by the malaria fire…Thin washed out by the terrible fever…they began to turn into new people” (as cited in Braslow, 1997, p. 79).

Of note, malaria therapy was invented in 1917 in Vienna (Wagner-Jauregg, 1927). This was the penultimate year of World War I. Wagner-Jauregg’s clinic, among others, “treated” World War I soldiers, suffering “war neurosis” (otherwise known as shell-shock), with painful electric current treatments, sometimes applied to the genitals (Bogousslavsky & Tatu, 2013; Eissler, 1986). After the war, Wagner-Jauregg was officially investigated for cruelty and torture. Although he was cleared, a subsequent re-analysis of the investigation casts serious doubt on the impartiality of the judicial process (Eissler, 1986). The shell-shock patients were called “shirkers” and “tremblers,” indicating the moral censure of such patients (Riebl & Sharp, 1992). The painful electric current “treatment” appears to be, in part, a punishment for the challenge these men represented to masculinity ideals, particularly in a nation at war (Mosse, 2000).

Many of the other somatic psychiatric treatments of the twentieth century were given predominantly to females. Pressman (1998) wrote that “an enduring mystery of the psychosurgery story, is why women were lobotomized nationally at a rate twice that of males” (p. 303). Braslow (1997) argued that the doctors entwined madness and unladylike behavior, and psychosurgery was seen as a potential intervention to restore femininity. In addition, women were “shackled, straightjacketed, bound and secluded” much more often than men (p. 157). Women who masturbated could be ordered to undergo clitoridectomy; men who masturbated and acted out “never lost their penises or testicles as a cure for these activities” (p. 168). In a feminist account, Showalter (1985) also portrayed psychiatry as a history of the colonization and subjugation of women.

Homosexuals’ bodies have also been a favored site for experimental twentieth century medical and surgical interventions in which treatment, social control, and punishment goals blur. Metrazol convulsive therapy, chemical castration with estrogens, surgical castration, clitoridectomy, brain operations, and aversive electrotherapy were all utilized with the aim to either convert the homoerotic desires to heterosexual ones or to obliterate desires all together (Murphy, 1992). Sometimes, as in the example of the British mathematician, Alan Turing, the treatment was accepted as an alternative to imprisonment (Hodges, 2012). It is of note that during the 1990s AIDS epidemic, which disproportionately impacted the male homosexual community, malaria therapy was resurrected. It was speculatively and unethically trialed as a potential cure for AIDS (Nierengarten, 2003).

My final historical example is the hormonal treatment of “tall girls” and “short boys.” From the 1960s through until the 1980s, large numbers of adolescents, who had no underlying medical pathology or hormonal abnormality, were prescribed hormonal treatment for their height (Cohen & Cosgrove, 2010). The reasons stated for such treatments, for what nowadays might be called “height dysphoria,” included that adolescents were distressed, and their height had negative social impact. For example, “careers in classical ballet or being an airline hostess were closed” to “tall girls” and their prospects of finding a husband were jeopardized (Wettenhall et al., 1975). Some adolescents and their parents eagerly sought this treatment, led to it by the encouragement of physicians and school nurses, enthusiastic media promotion, and pharmaceutical companies’ advertising. At the time, the hormones were declared safe, but years later concerns emerged about long-term adverse effects, including impaired fertility and increased risk of cancers (Benyi et al., 2014; Venn et al., 2004). Some boys treated with growth hormone developed Creutzfeld–Jacob disease, an aggressive early onset and fatal dementia. In Australia, this led to a federal government inquiry and an apology. In France, criminal charges were laid against some of the physicians involved (Cohen & Cosgrove, 2009, pp. 262, 350). Importantly, there had been a lack of controlled trials to confirm the efficacy, either on improving psychosocial outcome or the impact on height, of these treatments. A retrospective cohort study of “tall girls” revealed that 42% of the study group regretted the hormonal treatment they received (ibid., p. 232). Tall girls and short boys may be a visual affront to some societal “ideals” of male strength female fragility. This seems another part of the story of medicine acting to reinforce society’s sex stereotypes, and for some patients it came at disastrous personal cost.

## The Gender Affirmative Treatment Approach for Gender Dysphoric Youth: Medical Advance or Dangerous Medicine?

Masculinizing chest surgery is part of the controversial gender affirmative treatment approach to GD youth. This approach is underpinned by the view that a child or adolescent’s stated gender identity should be endorsed not questioned, and that they should be supported to undertake social transition, medical transition, masculinizing chest surgery, and, some also argue, genital surgery. Those advocating this approach consider these gender affirming treatments medically and ethically essential (Baams, 2021; de Vries et al., 2021; Transgender Health, 2020; Walch et al., 2021).

Others express concern about this approach (Biggs, 2020; D’Angelo et al., 2021; Levine, 2018, 2021; Malone, 2021). They note the limited and low-quality evidence base for the benefits, not only of mastectomy as I have done in this Letter, but for early social transition and the hormonal treatments for GD adolescents. Concerns are raised about the irreversible and long-term adverse impacts of these treatments on fertility and sexual function, as well as on bone, brain, and cardiovascular functioning. Concerns are expressed about the sharp, massive, and largely unexplained increase in GD youth, many with psychiatric and neurodevelopmental disorders, presenting to gender clinics. They caution against early social transition, hormonal, and surgical treatments of youth. Some ask: Why are these experimental interventions, with inherent risks and scarce, low-quality evidence for benefits, being implemented outside HREC regulated clinical trial settings?

The studies’ limitations also mean that regret and detransition rates are largely unknown. A widely cited study to support claims of less than 1% long-term regret rates is Wiepjes et al. (2018). However, this study only reported on stringently defined regret (as recorded by the clinician in the clinical record plus the prescription of sex hormones consistent with natal sex) in those who underwent gonadectomy, and then continued in follow-up (and 36% of the study population dropped out of follow-up). The adolescent subgroup was carefully selected, being meticulously assessed and managed via the rigorous “Dutch protocol” (de Vries & Cohen-Kettenis, 2012) and the vast majority had a relatively short follow-up period. Most contemporary youth gender clinics do not follow the “Dutch protocol,” which further limits the applicability of this study’s regret rate findings. It is worth highlighting that Keira Bell, a well-known detransitioner, would not meet the criteria to be categorized as a regret case in the Wiepjes et al. study as she never underwent gonadectomy. Celebratory stories of medically and surgically transitioned young people are regularly promoted by physicians and the media (Alcindor, 2015; Cohen, 2021) adding to the impression of overwhelming beneficial outcomes of this treatment approach. However, there are now increasing reports in the medical literature of regret and detransition (Entwistle, 2021; Littman, 2021; Pazos-Guerra et al., 2020; Vandenbussche, 2021) and these give us cause to question the claims of negligible regret rates.

In response to my historical examples, some may argue that informed consent and patient autonomy differentiates contemporary medicine from historical medicine. However, many of these historical treatments did require informed consent, either from the patient or a family member. In addition, informed consent is complex. A necessary condition for it is clinician honesty, which is not met if clinicians overstate the evidence base or act as “cheerleaders” for transition (Clayton et al., in press; Levine, 2019). The contemporary portrayal of the principle of patient autonomy as a simple solution for bioethical quandaries has been challenged by Mol (2008). Mol illustrated that the notion of “patient as a customer autonomously choosing health care” is simplistic. Patients’ choices are deeply entangled with other factors, including the influence of their clinicians and medical marketing. Some readers may also reject my discussing contemporary medicine for GD youth along with what they may consider as cruel historical treatments. However, we need to remember that, for the most part, these treatments were not judged as cruel at the time of use, were widely celebrated, and were implemented by well-intentioned physicians who fervently believed that they were helping their patients.

This is also a controversial topic outside the medical field. Contemporary feminist academics have divided views on transgender issues and the medicalization of GD youth (Gillis et al., 2007; Riley & Pearce, 2018; Stock, 2021). Thus, while some express full support, others express concern that the medicalization of such youth is reinforcing sexual stereotypes and gender binaries. They argue that a deeper analysis is required to understand why so many young people, particularly natal females, are now finding their natural bodies uninhabitable. Others are troubled with the fact that it is the powerful pharmaceutical and surgical industries that are creating trans-bodies.

Some influential LGBTIQ and parent groups are vocal in their support of affirmative treatments for GD youths, but other such groups express concern. Some consider that placing the label “trans” on gender non-conforming youth is an expression of the homophobia of our society. They are concerned that the hormonal and surgical interventions maybe a repetition, albeit unwittingly, of historical treatments that aimed at converting homosexual people to fit with heterosexual norms (Stock, 2021, pp. 83–84). There is emerging research data that support these concerns. Littman (2021), in her survey of 100 detransitioners, found 23% reported homophobia or difficulty accepting themselves as lesbian, gay or bisexual as a reason for their transition and subsequent detransition. The example of Iran is also often raised in this context. Jafari (2014) noted that Iran’s government boasts about their high rates of sexual reassignment surgeries as indicative of their commitment to human rights, and that some, including Western media sources, can portray the availability of such surgeries in Iran as liberal and progressive. Yet, in Iran, homosexuality is forbidden and punishable by the death penalty. To avoid this, homosexuals may have to undergo sexual reassignment surgery (see also Hamedani, 2014). Jafari (2014) commented that “ultimately the state’s goal–and the murkier side to this ‘liberal’ medico-legal development–is the assimilation of homosexual men and women within a binary gender paradigm” (p. 42). For more perspectives on the complexities of this situation in Iran and relevant cross-cultural issues, the interested reader is referred to Meyer (2016) and Sadjadi (2019).

## Conclusion

How, then, do we best read the affirmative treatment approach for GD youth? Should it be read triumphantly as cutting-edge, ethical, and evidence-based medicine continuing on its progressive march of improving human life? Or is it a manifestation of dangerous medicine, that despite best intentions will cause more harm than benefit to vulnerable youths, and over which future historians and physicians will shake their heads?

Between the ages of 16 and 20, Kiera Bell identified as a man and took testosterone and underwent a double mastectomy. She then detransitioned. In a court testimony, she described her regret: “I felt like a fraud…more lost, isolated and confused than I did when I was pre-transitioned…only recently…I have started to think about having children and if that is ever a possibility, I have to live with the fact that I will not be able to breastfeed my children…I made a brash decision as a teenager…trying to find confidence and happiness…now the rest of my life will be negatively affected” (Bell, 2020, pp. 21–22). In these words, Bell holds herself responsible for making a “brash decision” in her youth. This may be an indication of maturity and taking responsibility, but it also has a more concerning element–a victim blaming herself for mistreatment. In my view, the medical profession needs to consider whether, in its championing of the gender affirmative approach for GD youth, it is also acting brashly and making mistakes that will negatively impact some young people for the rest of their lives.

## References

[CR1] Alcindor, Y. (2015, March 14). Transgender teen Jazz Jennings lands clean and clear campaign. *USA Today.* Retrieved from https://www.usatoday.com

[CR2] Austin SC, Stolley PD, Lasky T (1992). The history of malariotherapy for neurosyphilis, modern parallels. Journal of the American Medical Association.

[CR3] Baams L (2021). Equity in paediatric care for sexual and gender minority adolescents. The Lancet Child & Adolescent Health.

[CR4] Bell, K. (2020). *British High Court Case No: CO/60/2020 Bell v Tavistock* (01/12/2020). Retrieved from https://www.judiciary.uk

[CR5] Benyi E, Kieler H, Linder M, Ritzén M, Carlstedt-Duke J, Tuvemo T, Westphal O, Sävendahl L (2014). Risks of malignant and non-malignant tumours in tall woman treated with high dose oestrogen during adolescence. Hormone Research in Pediatrics.

[CR6] Biggs M (2020). Gender dysphoria and psychological functioning in adolescents treated with GnRHa: Comparing Dutch and English prospective studies [Letter to the Editor]. Archives of Sexual Behavior.

[CR7] Bogousslavsky J, Tatu L (2013). French neuropsychiatry in the Great War: Between moral support and electricity. Journal of the History of the Neurosciences.

[CR8] Brandt AM (1985). No magic bullet: A social history of venereal disease in the United States since 1880.

[CR9] Braslow J (1997). Mental ills and bodily cures: Psychiatric treatment in the first half of the twentieth century.

[CR10] Clayton, A., Malone, W., Clarke, P., Mason, J., & D’Angelo, R. (in press). The signal and the noise: Questioning the benefits of puberty blockers for gender dysphoria–a commentary on Rew et al. (2021). *Child and Adolescent Mental Health.* 10.1111/camh.12533 34936180

[CR11] Cohen, J. (Producer). (2021). *Australian story: A balancing act/Michelle Telfer* [Television broadcast]. Australian Broadcasting Commission. Retrieved from abc.net.au

[CR12] Cohen, S., & Cosgrove, C. (2009). *Normal at any cost: Tall girls, short boys and the medical industry’s quest to manipulate height*. New York: Jeremy P. Tarcher/Penguin.

[CR13] Cohen S, Cosgrove C (2010). Too tall, too small? The temptation to tinker with a child’s height. The Lancet.

[CR14] Cohen WA, Shah NR, Iwanicki M, Therattil PJ, Keith JD (2019). Female-to-male transgender chest contouring: A systematic review of outcomes and knowledge gaps. Annals of Plastic Surgery.

[CR15] D’Angelo R, Syrulnik E, Ayad S, Marchiano L, Kenny DT, Clarke P (2021). One size does not fit all: In support of psychotherapy for gender dysphoria [Letter to the Editor]. Archives of Sexual Behavior.

[CR16] Davis G (2008). The cruel madness of love: Sex, syphilis and psychiatry in Scotland, 1880–1930.

[CR17] de Vries ALC, Cohen-Kettenis PT (2012). Clinical management of gender dysphoria in children and adolescents: The Dutch approach. Journal of Homosexuality.

[CR18] de Vries ALC, Richards C, Tishelman AC, Motmans J, Hannema SE, Green J, Rosenthal SM (2021). Bell v Tavistock and Portman NHS Foundation Trust [2020] EWHC 3274: Weighing current knowledge and uncertainties in decisions about gender-related treatment for transgender adolescents. International Journal of Transgender Health.

[CR19] Diefenbach GJ, Diefenbach D, Baumeister A, West M (1999). Portrayal of lobotomy in the popular press: 1935–1960. Journal of the History of the Neurosciences.

[CR20] “Doctor” Mosquito. (1926). *Newcastle morning Herald and Miners’ Advocate*, p. 8. Retrieved from trove.nla.gov.au

[CR21] Ellery, R. S. (1956). *The cow jumped over the moon: Private papers of a psychiatrist.* Melbourne: F.W. Cheshire.

[CR22] Eissler KR (1986). Freud as an expert witness: The discussion of war neuroses between Freud and Wagner-Jauregg.

[CR23] Entwistle K (2021). Debate: Reality check–Detransitioner’s testimonies require us to rethink gender dysphoria. Child and Adolescent Mental Health.

[CR24] Frankenburg FR, Baldessarini RJ (2008). Neurosyphilis, malaria and the discovery of antipsychotic agents. Harvard Review of Psychiatry.

[CR25] Freeman W (1961). Adolescents in distress: Therapeutic possibilities of lobotomy. Diseases of the Nervous System.

[CR26] Gillis S, Howie G, Munford R (2007). Third wave feminism: A critical exploration.

[CR27] Godding WW (1897). Active treatment in general paralysis of the insane. British Medical Journal.

[CR28] Grob GN (1994). The mad among us: A history of the care of America’s mentally ill.

[CR29] Hamedani, A. (2014). The gay people pushed to change their gender. *BBC News.* Retrieved from https://from www.bbc.com/news/magazine-29832690

[CR30] Harrington A (2019). Mind fixers: Psychiatry’s troubled search for the biology of mental illness.

[CR31] Hodges A (2012). Alan Turing: The enigma.

[CR32] Hurn, J. D. (1998). *The history of general paralysis of the insane in Britain, 1830–1950.* Unpublished doctoral thesis, University of London.

[CR33] Jafari F (2014). Transsexuality under surveillance in Iran: Clerical control of Khomeini’s fatwas. Journal of Middle East Women’s Studies.

[CR34] Kuper LE, Rider GN, St Amand CM (2021). Recognizing the importance of chest surgery for transmasculine youth. Pediatrics.

[CR35] Kuper LE, Stewart S, Preston S, Lau M, Lopez X (2020). Body dissatisfaction and mental health outcomes of youth on gender-affirming hormone therapy. Pediatrics.

[CR36] Lehmann, C. (2021). Trans-‘inclusive’ language is erasing women’s biology. *The Australian.* Retrieved from theaustralian.com.au

[CR37] Lerner BH, Caplan AL (2016). Judging the past: How history should inform bioethics. Annals of Internal Medicine.

[CR38] Levine SB (2018). Ethical concerns about emerging treatment paradigms for gender dysphoria. Journal of Sex and Marital Therapy.

[CR39] Levine SB (2019). Informed consent for transgender patients. Journal of Sex & Marital Therapy.

[CR40] Levine SB (2021). Reflections on the clinician’s role with individuals who self-identify as transgender. Archives of Sexual Behavior.

[CR41] Littman L (2021). Individuals treated for gender dysphoria with medical and/or surgical transition who subsequently detransitioned: A survey of 100 detransitioners. Archives of Sexual Behavior.

[CR42] McDougall R, Notini L, Delany C, Telfer M, Pang KC (2021). Should clinicians make chest surgery available to transgender male adolescents?. Bioethics.

[CR43] Malone, W. (2021). Time to hit pause on ‘pausing’ puberty in gender-dysphoric youth. *Medscape.* Retrieved from medscape.com

[CR44] Marinkovic M, Newfield RS (2017). Chest reconstructive surgeries in transmasculine youth: Experience from one pediatric center. International Journal of Transgenderism.

[CR45] Mehringer JE, Harrison JB, Quain KM, Shea JA, Hawkins LA, Dowshen NL (2021). Experience of chest dysphoria and masculinizing chest surgery in transmasculine youth. Pediatrics.

[CR46] Meyer LD (2016). Book forum: Professing selves: Transsexuality and same-sex desire in contemporary Iran by Afsaneh Najmabadi (review). Journal of Women’s History.

[CR80] Milrod, C., & Karasic, D. H. (2017). Age is just a number: WPATH-affiliated surgeons’ experiences and attitudes towards vaginoplasty in transgender females under 18 years of age in the United States. *Journal of Sexual Medicine*, *14*, 624–634. 10.1016/j.jsxm.2017.02.007 10.1016/j.jsxm.2017.02.007 28325535

[CR47] Mol A (2008). The logic of care: Health and the problem of patient choice.

[CR48] Mosse, G. L. (2000). Shell-shock as a social disease. *Journal of Contemporary History, 35*(1), 101–108. https://www.jstor.org/stable/261184

[CR49] Murphy TF (1992). Redirecting sexual orientations: Techniques and justifications. Journal of Sex Research.

[CR50] Nierengarten MB (2003). Malariotherapy to treat HIV patients?. The Lancet Infectious Diseases.

[CR51] Olson-Kennedy, J. (2015). *The future of trans care in the new millennium*. Gender Infinity Annual Conference. Retrieved from https://youtu.be/pO8v--tztSg

[CR52] Olson-Kennedy J, Warus J, Okonta V, Belzer M, Clark LF (2018). Chest reconstruction and chest dysphoria in transmasculine minors and young adults: Comparisons of nonsurgical and postsurgical cohorts. JAMA Pediatrics.

[CR53] Pazos Guerra M, Gómez Balaguer M, Gomes Porras M, Hurtado Murillo F, Solá Izquierdo E, Morillas Ariño C (2020). Transexualidad: Transiciones, detransiciones y arrepentimientos en España [Transexuality: Transitions, detransitions and regrets in Spain]. Endocrinología, Diabetes y Nutrición.

[CR54] Pressman JD (1998). Last resort: Psychosurgery and the limits of medicine.

[CR55] Quétel C (1990). History of syphilis.

[CR56] Reverby SM, Baylis F, Dreger A (2018). “So what?”: Historical contingency, activism, and reflections on the studies in Tuskegee and Guatemala. Bioethics in action.

[CR57] Riebl L, Sharp P (1992). Julius Wagner von Jauregg: A reappraisal. Australian and New Zealand Journal of Psychiatry.

[CR58] Riley C, Pearce L (2018). Feminism and women’s writing: An introduction.

[CR59] Sadjadi S (2019). Deep in the brain: Identity and authenticity in pediatric gender transition. Cultural Anthropology.

[CR60] Sadowsky J (2017). Electroconvulsive therapy in America: The anatomy of a medical controversy.

[CR61] Scull A (2015). Madness in civilization: A cultural history of insanity from the Bible to Freud, from the madhouse to modern medicine.

[CR62] Shaw, B. (1929). In: General paralysis, a report of a meeting of the General Paralysis Subcommittee of the Royal Medico-Psychological Association. *Journal of Mental Science, 308*, 1–30.

[CR63] Showalter E (1985). The female malady: Women, madness and English culture, 1830–1980.

[CR64] Stock K (2021). Material girls: Why reality matters for feminism.

[CR65] Swain K (2018). ‘Extraordinarily arduous and fraught with danger’: Syphilis, Salvarsan, and general paresis of the insane. The Lancet Psychiatry.

[CR66] Telfer, M. M. (2019). Witness statement of Associate Professor Michelle Telfer. *Royal Commission into Victoria’s mental health system.* Retrieved from: https://rcvmhs.archive.royal commission.vic.gov.au

[CR67] Tollit MA, May T, Maloof T, Telfer MM, Chew D, Engel M, Pang K (2021). The clinical profile of patients attending a large, Australian pediatric gender service: A 10-year review. International Journal of Transgender Health.

[CR68] Transgender Health: An Endocrine Society position statement. (2020, December 15). Retrieved from https://www.endocrine.org/advocacy/position-statements/ transgender-health.

[CR69] Valenstein ES (1986). Great and desperate cures: The rise and decline of psychosurgery and other radical treatments for mental illness.

[CR70] van de Grift TC, Kreukels BPC, Elfering L, Özer M, Bouman M-B, Buncamper ME, Smit JM, Mullender MG (2016). Body image in transmen: Multidimensional measurement and the effects of mastectomy. Journal of Sexual Medicine.

[CR71] Vandenbussche E (2021). Detransition-related needs and support: A cross-sectional online survey. Journal of Homosexuality.

[CR72] Venn A, Bruinsma F, Werther G, Pyett P, Baird D, Jones P, Rayner J, Lumley J (2004). Oestrogen treatment to reduce the adult height of tall girls: Long-term effects on fertility. The Lancet.

[CR73] Wagner-Jauregg, J. (1927). *Nobel lecture: The treatment of dementia paralytica by malaria inoculation.* Retrieved from https://www.nobelprize.org/prizes/medicine/1927/wagner-jauregg/lecture/

[CR74] Walch A, Davidge-Pitts C, Safer JD, Lopez X, Tangpricha V, Iwamoto SJ (2021). Proper care of transgender and gender diverse persons in the setting of proposed discrimination: A policy perspective. Journal of Clinical Endocrinology and Metabolism.

[CR75] Wettenhall HN, Cahill C, Roche AF (1975). Tall girls: A survey of 15 years management and treatment. Journal of Pediatrics.

[CR76] Wiepjes CM, Nota NM, de Blok CJM, Klaver M, de Vries ALC, Wensing-Kruger SA, de Jongh RT, Bouman M-B, Steensma TD, Cohen-Kettenis P, Gooren LJG, Kreukels BPC, den Heijer M (2018). The Amsterdam Cohort of Gender Dysphoria Study (1972–2015): Trends in prevalence, treatment, and regrets. Journal of Sexual Medicine.

